# Munchausen syndrome in the emergency department mostly difficult, sometimes easy to diagnose: a case report and review of the literature

**DOI:** 10.1186/1749-7922-4-38

**Published:** 2009-11-12

**Authors:** Rinaldo Lauwers, Nele Van De Winkel, Nathalie Vanderbruggen, Ives Hubloue

**Affiliations:** 1 Emergency Department UZ Brussel, Brussels, Belgium; 2 Department of Surgery UZ Brussel, Brussels, Belgium; 3 Department of Psychiatry UZ Brussel, Brussels, Belgium

## Abstract

Munchausen syndrome is a rare psychiatric disorder in which patients inflict on themselves an illness or injury for the primary purpose of assuming the sick role. Because these patients can present with many different complaints and clinical symptoms, diagnosis is often made at a later stage of hospitalisation. In contrast we report a case of a 40-year old woman very easy to diagnose with Munchausen syndrome.

This trained nurse presented at our emergency department (ED) complaining of abdominal pain. Interviewed by the medical trainee, she immediately confessed having put a knitting needle into her urethra four days earlier. She was not able to remove it anymore because it was beyond her reach. Abdominal X-ray confirmed the presence of the needle and a median laparotomy was performed to remove it. The diagnosis of Munchausen syndrome seemed immediately obvious in this case.

## Introduction

Doctors working at the emergency department often encounter patients who exaggerate, feign or aggravate their symptoms in order to get more attention and be treated more rapidly. In Munchausen syndrome, a particular form of factitious disorders, symptoms of illness or injury are intentionally produced for psychological reasons in order to be hospitalised and even to submit her to invasive interventions [1]. Many psychiatric disorders are seen at the ED, from depressive patients over psychosomatic complaints to severe psychiatric disorders as there are Munchausen syndrome, conversion disorders, hypochondriasis, malignering and somatisation disorders. The lack of medical documentation to substantiate the self-reported medical history is notable and good physical examination (scars, little haemorrhages) is indispensable and can help to diagnose more rapidly Munchausen syndrome which isn't easy in the ED.

## Case Report

**Figure 1:**
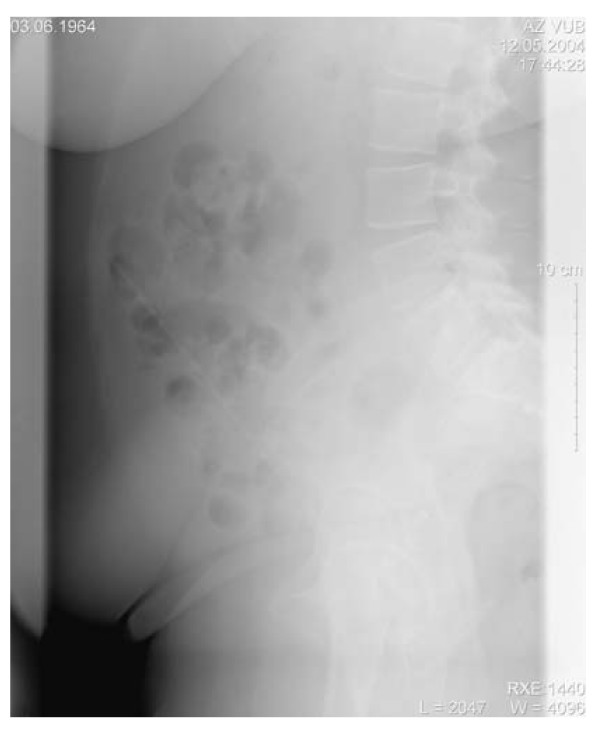
**An abdominal X-ray confirmed the diagnosis of an intra abdominal foreign body**.

**Figure 2:**
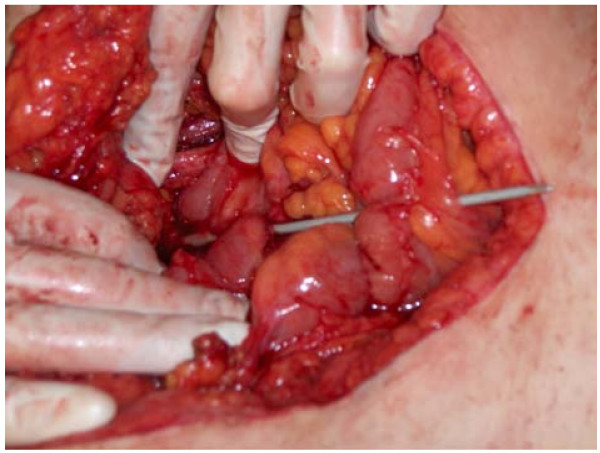
**Knitting needle perforations to the bladder, small intestine and colon transversum**.

A 40-year-old female presented at the ED triage desk with abdominal pain without any further complaints. Interviewed by a medical student she admitted having put a knitting needle into her urethra repetitively for the last 4 days and that now the needle was beyond her reach. Further interrogation was not contributively and except for abdominal tenderness physical examination was normal with initial vital signs of BP 124/76 mmHg, heart rate 91 bpm, a respiratory rate of 10 breaths per min, and temperature of 36.8°C. Complementary investigations were performed, the CBC revealed hematocrit 31% (36.4 - 43.9), WBC 11.0 × 10^3^/mm^3 ^(3.6 - 9.6) and the chemistry panel showed c-reactive protein 38.5 mg/L (< 5) as abnormal values. An abdominal X-ray confirmed the diagnosis of an intra abdominal foreign body (fig. 1). After multidisciplinary consult a median laparotomy was performed under epidural assisted general anesthesia. In the operating field we saw that the knitting needle had perforated the bladder, small intestine and colon transversum (fig. 2). Inspection of the needle revealed that the top had been sharpened. The needle was removed gently by pulling it out starting from the bladder, closing each perforation without resection of the intestine. Fibrinogenic composit around the needle made us conclude that the patient had been putting in the needle for a longer period than the four days she revealed. After one night in the recovery room she was discharged to the ward where she started eating the next day.

Psychiatric consult, done before the operation, concluded at a diagnosis of Munchausen syndrome. In her childhood the patient apparently didn't get much attention until she was admitted to the hospital for an acute appendicitis. The support from her family during that period of illness was so emotionally warming that she started to injure herself for the primary purpose of assuming the sick role.

The medical history revealed one former stay in our hospital with a diagnosis of urosepsis and bladder abces, without any causal pathogene and a suspicion of a psychiatric disorder. This was however never investigated since the patient left hospital when a psychiatirc consult was proposed. During her current stay she also admitted to have contaminated the fistula which developed due to the bladder abces for months so that it would not cure Unfortunatelly she again resigned psychiatric help against medical advise on this stay and left hospital after a couple of days.

## Discussion and Review of Literature

Factitious disorders are particularly challenging and fascinating at the ED where triage according to severity of illness and quick diagnosis are key issues for efficacy. Intentionally exaggerated, feigned, simulated, aggravated, or self-induced illnesses are most of the time frustrating for ED personel but can be very exhausting to diagnose. The name of Munchausen syndrome, referring to the historical figure of Baron Karl von Munchausen (1720-1797) was first applied to a psychiatric disorder in 1951 by Asher, discribing patients with neurological, haematologic and gastrointestinal disorders [2]. Patients with Munchausen syndrome often have co-morbid severe personality disorders, but the link with the primary syndrome is unclear. According to the American Psychiatric Association the DSM-IV TR criteria for factitious disorders are [3]:

1. Intentional production or feigning of psychological or physical signs or symptoms

2. Assumption of the sick role as motivation for the behavior

3. Absence of external gain, such as avoiding legal responsibility or improving physical wellbeing, as in malingering.

The following subtypes are specified

1. Patients with primarily physical signs and symptoms

2. Patients with primarily psychological signs and symptoms

3. Mixed subtype

A high index of suspicion is necessary whenever the characteristic presenting features essential in recognizing Munchausen syndrome are present [4]:

Essential features

Pathologic lying (pseudologia fantastica)

Peregrination (traveling or wandering)

Recurrent, feigned or simulated illness

Supporting features

Borderline and/or antisocial personality traits

Deprivation in childhood

Equanimity for diagnostic procedures

Equanimity for treatments or operations

Evidence of self-induced physical signs

Knowledge of or experience in the medical field

Male gender

Multiple hospitalizations

Multiple scars (usually abdominal)

Police record

Unusual or dramatic presentation

In our patient the diagnosis of Munchausen syndrome was very easy due to the presence of the needle in a cooperative patient and immediate psychiatric consult that revealed 2 out of 3 essential features and multiple of the supporting features. Mostly diagnosis is very difficult in the ED and should be a diagnosis of exclusion.

The prevalence of Munchausen syndrome is rare but most patients presenting with the disorder are admitted to hospital through the ED because of the dramatic presentation of an apparently severe illnesses [5]. The potential for significant inadvertent morbidity and mortality exists; in our patient the needle could have caused a perforation of the aorta or other organs. Further diagnostic procedures and treatment interventions can also cause more morbidity or mortallity, by the intervention itself or through the patients contribution (eg. taking anticoagulant drugs). In contrast to this case most of the Munchausen syndrome present in males and incidence peaks in young-to-middle-aged adults, mostly moving to different physicians and hospitals repeatedly simulating or self-inducing a single medical problem or with a wide diversity of medical problems leading to a lack of medical documentation to substantiate the self-reported medical history [6]. Physical examination can be very contributive in checking patients history but not in diagnosis because the great mimicking capacity of the subject to generate physical findings and symptoms.

Although our patient asked for a psychiatric interview most patients are seldom willing to admit that they have feigned or caused their own medical problems. After treatment of the selfinduced disease, patients mostly discharge against medical advice because they are afraid that truth will come above, or start lying resulting in chronic lying behaviour.

Differential diagnosis with other psychiatric disorders must be made. Conversion disorders, hypochondriasis, malingering, somatisation disorders and Munchausen by proxi are to be considered. The patient suffering Munchausen syndrome or Munchausen by proxy (mostly children) have no clear gain and Munchausen patients actively seek hospitalization and invasive painful procedures simply to undergo them, whereas in self-mutilation the injury is intended to assist the individual in dissociating from immediate tension. Cause and pathophysiology remain unclear and the prevalence of factitious disorders is probably in the range of 0.2-1% of hospital inpatients.

Treatment of Munchausen syndrome primarily consist in treating the self-induced illness before psychiatric help can be given. Unfortunately most patients refuse psychiatric help and leave hospital even before correct diagnosis is made [7].

## Conclusion

In such a difficult matter as emergency medicine where rapid diagnosis and installation of treatment are key-points, every ED doctor encounters funny, bizarre or puzzling stories. Diagnosis of Munchausen syndrome is seldom as easy as it was for us. In our opinion we can not expect that the diagnosis of Munchausen syndrome is made at the ED where initial care, stabilization and treatment of patients is the first issue. If suspicion of a factitous disorder exists psychiatric consultation and referral should be offered even if the patient declines. Because most patients leave hospital after discharge against medical advice and present in another hospital with the same or other symptoms, it could be interesting that a database was created for this disorder.

## Competing interests

The authors declare that they have no competing interests.

## Authors' contributions

RL: emergency doctor who received the patient and put her a sleep during the surgery, NVDW: surgeon on duty who performed the laparotomy, NV: psychiatrist on duty IH head of the ED

## Consent section

Written informed consent was obtained from the patient for publication of this case report and accompanying images. A copy of the written consent is available for review by the Editor-in-Chief of this journal.
